# Retraction Note: LINC01354/microRNA-216b/KRAS Axis Promotes the Occurrence and Metastasis of Endometrial Cancer

**DOI:** 10.1186/s11671-023-03831-7

**Published:** 2023-03-20

**Authors:** Yan Zhang, Wei Zhao, Fei Na, Meng Li, Shengchun Tong

**Affiliations:** Department of Gynecology, The Fourth Affiliated Hospital of China Medical University, No. 4, Chongshan East Road, Huanggu District, Shenyang, 110032 China

**Retraction note: Nanoscale Research Letter (2021) 17:21**

10.1186/s11671-021-03640-w

The Editors in Chief have retracted this article. After publication, concerns were raised regarding lack of scale for tumours in Fig. 6A and poor quality of Western blots. Furthermore, ECC-1 cell line has been reported as contaminated by Ishikawa. The authors did not respond to requests to supply raw data or the ethics permit. The Editors, therefore, have lost confidence in the integrity of the article's findings. The authors did not respond to any correspondence from the Editors about this retraction.

